# Cationic Surface Modification Combined with Collagen Enhances the Stability and Delivery of Magnetosomes for Tumor Hyperthermia

**DOI:** 10.3390/jfb16120461

**Published:** 2025-12-12

**Authors:** Yu Wang, Conghao Lin, Yubing Zhang, Wenjun Li, Hongli Cui, Bohan Li, Zhengyi Liu, Kang Wang, Qi Wang, Yinchu Wang, Kangning Lv, Yandi Huang, Hongqin Zhuang, Song Qin

**Affiliations:** 1 Key Laboratory of Coastal Biology and Biological Resource Utilization, Yantai Institute of Coastal Zone Research, Chinese Academy of Sciences, Yantai 264003, China; wangyu209@mails.ucas.ac.cn (Y.W.); wjli@yic.ac.cn (W.L.); hlcui@yic.ac.cn (H.C.); zyliu@yic.ac.cn (Z.L.); kangwang@yic.ac.cn (K.W.); sdlywangqi@163.com (Q.W.); ycwang@yic.ac.cn (Y.W.); 2 University of Chinese Academy of Sciences, Beijing 100049, China; 3 State Key Laboratory of Bioelectronics, National Demonstration Center for Experimental Biomedical Engineering Education, School of Biological Science and Medical Engineering, Southeast University, Nanjing 210096, China; 230258335@seu.edu.cn; 4 The State Key Laboratory of Pharmaceutical Biotechnology, School of Life Sciences, Nanjing University, Nanjing 210023, China; 602023300062@smail.nju.edu.cn; 5 Featured Laboratory for Biosynthesis and Target Discovery of Active Components of Traditional Chinese Medicine, School of Traditional Chinese Medicine, Binzhou Medical University, Yantai 264003, China; bohanli@bzmc.edu.cn; 6 State Key Laboratory of Heavy Oil Processing and Center for Bioengineering and Biotechnology, College of Chemistry and Chemical Engineering, China University of Petroleum (East China), Qingdao 266580, China; bz23030029@s.upc.edu.cn; 7 School of Life Sciences, Yantai University, Yantai 264003, China; 201970503202@s.ytu.edu.cn

**Keywords:** cationic modification, collagen, delivery efficiency, magnetosomes, suspension stability, tumor magnetic hyperthermia

## Abstract

Magnetosomes (MTS), membrane-enclosed magnetic nanoparticles naturally biomineralized by magnetotactic bacteria, are promising materials for tumor hyperthermia owing to their good biocompatibility and heating efficiency. However, their application is limited by poor suspension stability and low injectability at high concentrations. This study aimed to enhance magnetosome stability and delivery performance through surface cationization combined with collagen matrix stabilization. The resulting cationic magnetosomes (CMTS) exhibited an increased positive charge on the outer membrane. Collagen, functioning as a negatively charged matrix under mildly alkaline conditions, effectively stabilized the cationic magnetosomes, forming CMTS–collagen aqueous suspensions (CMTS-Colas) that remained well-suspended for over 24 h and could be easily resuspended after 10 days of storage. Compared with native magnetosome suspensions, CMTS in collagen displayed smaller hydrodynamic diameters and significantly improved injectability through 26G and 31G fine needles. Under an alternating magnetic field, 2 mg/mL CMTS-Colas efficiently induced over 98% apoptosis in hepatoma cells after two treatment sessions and led to complete loss of cell viability after three sessions. These findings demonstrate that CMTS-Colas substantially improve the suspension stability and injectability of magnetosomes while maintaining strong hyperthermic efficacy, suggesting a promising strategy for stabilizing magnetosomes and potentially benefiting other charged, aggregation-prone magnetic biomaterials.

## 1. Introduction

Cancer remains a major global health challenge, with approximately 20 million new cases and 10 million deaths reported in 2022 [1,2]. Magnetic hyperthermia therapy (MHT) has emerged as a promising alternative to conventional tumor treatments due to its minimal invasiveness, high targeting precision, and immunomodulatory effects. By delivering hyperthermia agents to tumor sites and applying a high-frequency alternating magnetic field (HF-AMF), MHT induces localized heating (e.g., 40–46 °C), selectively damaging tumor tissues while preserving healthy cells [3,4]. Furthermore, thermal stimulation can trigger immunogenic cell death by promoting the release and surface exposure of damage-associated molecular patterns, thus facilitating immune recognition and elimination of tumor cells [5,6].

Chemically synthesized iron oxide nanoparticles (SIONP) are commonly used as MHT agents, but their relatively low magnetothermal conversion efficiency often necessitates high doses, which may increase the risk of side effects and complicate clinical applications [7,8]. To address this issue, various strategies have been explored, such as particle size enlargement, heavy metal doping, morphological tuning, and surface functionalization [9,10,11].

As a biologically derived alternative to SIONP, magnetosomes (MTS) have attracted growing interest. MTS typically comprise magnetite (Fe_3_O_4_) cores biomineralized by magnetotactic bacteria and naturally encapsulated within biological membranes. Owing to their uniform size distribution and biologically regulated single-domain structure, MTS are among the most efficient hyperthermia agents [8,12]. In addition, MTS exhibit comparable cytotoxicity to SIONP in vitro and have demonstrated good biocompatibility in animal models following intravenous or intratumoral injection [13,14,15]. These attributes highlight the potential of MTS for hyperthermia applications and underscore their prospects for future clinical translation.

Inspired by the NanoTherm^®^ Therapy System—an aminosilane-coated iron oxide nanoparticle colloidal suspension delivered percutaneously into tumor tissue and the first and currently only hyperthermia agent approved by the European Medicines Agency for glioblastoma treatment—intratumoral injection is considered the most feasible route for the clinical translation of magnetosome-based hyperthermia [16]. Although the biological membrane is considered to play an important role in stabilizing MTS aqueous suspensions [17], it is often partially removed to reduce endotoxin, which compromises the stability of the formulations. As a result, high-concentration suspensions (e.g., 1 mg/mL) tend to aggregate into large particles and rapidly sediment, often within 10 min after homogenization. Surface coating with positively charged poly-L-lysine can delay sedimentation but only extends the dispersion time to approximately 20 min at 1 mg/mL [14]. Such instability poses several challenges for hyperthermia application and clinical translation: aggregation and sedimentation disrupt uniform syringe distribution, undermine quantitative precision, constrain injection timing, and increase the risk of needle blockage in minimally invasive procedures. Therefore, stabilizing MTS suspensions is essential for ensuring their effectiveness and practical application in hyperthermia treatments.

**Figure 1:**
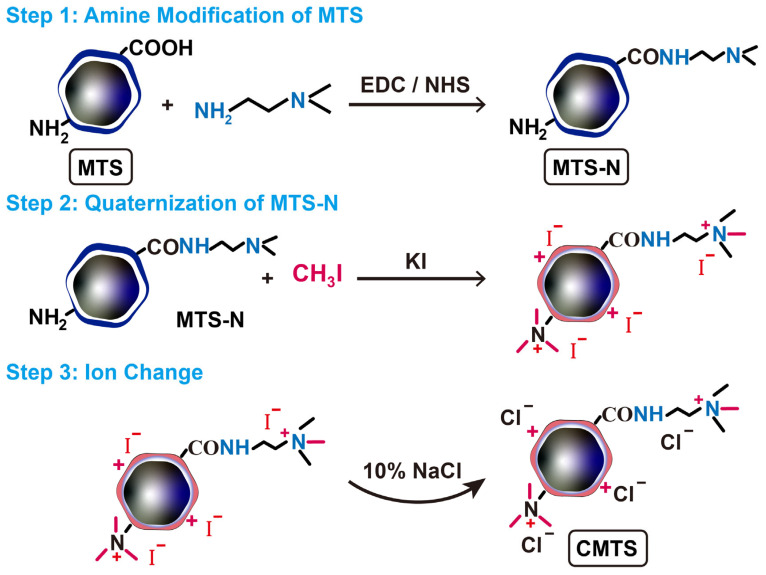
Schematic illustration of the surface modification and synthesis of CMTS.

In this study, cationized magnetosomes (CMTS) were developed by modifying surface carboxyl (COOH) and primary amine (NH_2_) groups present on the biological membrane of native MTS, resulting in a stable, positively charged surface under mildly alkaline conditions (Figure 1). To further improve suspension stability, medical-grade type I collagen was introduced as a biocompatible, negatively charged matrix. To the best of our knowledge, this is the first time that collagen has been used to stabilize MTS. The introduction of a collagen matrix resulted in the formation of CMTS-collagen aqueous suspensions (CMTS-Colas), which not only enhanced the stability of MTS but also improved their delivery performance. At 20 mg/mL—the highest MTS concentration reported for injection to our knowledge—CMTS-Colas remained well-dispersed for at least 24 h, representing a substantial improvement in suspension stability over previous formulations. Compared with native MTS suspensions, CMTS-Colas exhibited significantly improved syringe-based delivery through fine-gauge needles. Furthermore, CMTS-Colas proved efficacious in in vitro hyperthermia, reaching therapeutic temperatures and significantly reducing hepatoma cell viability.

## 2. Materials and Methods

### 2.1. Materials

*Magnetospirillum magneticum* strain AMB-1 (AMB-1) was obtained from the American Type Culture Collection. 1-(3-Dimethylaminopropyl)-3-ethylcarbodiimide hydrochloride (EDC·HCl), 1-Hydroxypyrrolidine-2,5-dione (NHS), and *N*,*N*-dimethylethylenediamine were purchased from Shanghai Bidepharm Co., Ltd. (Shanghai, China). Medical-grade type I collagen solution (tilapia-derived, sterile, 1 mg/mL, MW ~300 kDa) was kindly provided by Yantai LongStrong Biotechnology Co., Ltd. (Yantai, China). The Kinetic Chromogenic LAL Assay (KCA) kit and associated reagents were from Zhanjiang A&C Biological Ltd. (Zhanjiang, China). Syringes with 26G (0.45 × 12 mm) and 31G (0.25 × 8 mm) needles were obtained from Shandong Ande Healthcare Apparatus Co., Ltd. (Zibo, China) and Promisemed Medical Devices Inc. (Hangzhou, China), respectively. Cell culture consumables (e.g., flasks, multi-well plates) were purchased from Corning Inc. (Corning, NY, USA). Reagents for AMB-1 culture and MTS production followed our previous study [18]. Unless otherwise specified, other chemicals were obtained from Macklin Biochemical Co., Ltd. (Shanghai, China).

Mouse fibroblast cells (L929), human umbilical vascular endothelial cells (HUVEC), mouse hepatoma cells (H22), human hepatoma cells (HepG2), fetal bovine serum (FBS), 100× penicillin-streptomycin (P/S) solution, phosphate-buffered saline (PBS), and trypsin were obtained from Wuhan Pricella Biotechnology Co., Ltd. (Wuhan, China). High-glucose Dulbecco’s Modified Eagle Medium (DMEM) was purchased from Wuhan Servicebio Technology Co., Ltd. (Wuhan, China). DAPI fluorescent staining solution was obtained from Solarbio Science & Technology Co., Ltd. (Beijing, China). The Cell Counting Kit-8 (CCK-8) and Annexin V-FITC/PI apoptosis detection kit were from MeilunBio Co., Ltd. (Dalian, China).

### 2.2. Cultivation of AMB-1 and MTS Extraction

The composition and preparation procedure of all culture media followed our previously established protocol, under which AMB-1 was also cultivated. The simplified fermentation procedure and optimized iron supplementation strategy developed in that study were also adopted to yield MTS in flask fermentation [18].

Following flask fermentation, the culture was concentrated using a tangential flow filtration system (Viva Flow 200, Sartorius, Bohemia, NY, USA), and cells were harvested by centrifugation at 15,000× *g*. The resulting pellet was resuspended in 0.1 mol/L NaOH, heated to 60 °C under nitrogen protection, and stirred for 1 h to lyse the cells and release MTS. A neodymium magnet (~300 mT) was applied externally to separate MTS from cell debris. The crude MTS were then resuspended in 0.1 mol/L NaOH, heated to 50 °C under nitrogen protection with continuous stirring for 15 min, and magnetically separated. This alkaline washing step was repeated three more times to further reduce residual endotoxins. Finally, MTS were washed with distilled water and freeze-dried. Endotoxin in the purified MTS was quantified using the KCA kit.

### 2.3. Transmission Electron Microscope (TEM) of AMB-1 and MTS

A 1 mL aliquot of cultured AMB-1 was collected by centrifugation at 5000× *g*. The harvested cells and 50 µg of MTS were each resuspended in 1 mL distilled water. Then, 5 µL of each suspension was dropped onto a copper grid, air-dried under clean laminar flow, and examined by TEM (JEM-1400, JEOL, Tokyo, Japan) for morphological analysis of AMB-1 cells and isolated MTS.

### 2.4. Preparation of CMTS

A total of 300 mg of MTS was dispersed in 25 mL distilled water and stirred at 600 rpm. A solution of 70 mg *N*,*N*-Dimethylethylenediamine, 150 mg EDC·HCl, and 135 mg NHS dissolved in 5 mL distilled water was added. The pH was adjusted to 6.0–6.5 and stirred at room temperature for 1 h, then adjusted to 7.5–8.0 and stirred for an additional hour. Afterward, the mixture was centrifuged at 3000× *g*, and the residue was washed three times with distilled water to obtain amine-modified magnetosomes (MTS-N).

MTS-N was then resuspended in 30 mL distilled water, followed by addition of 300 mg KI and 1000 µL methyl iodide. The pH was adjusted to approximately 11, and the reaction mixture was stirred under nitrogen at room temperature for 1 h. Subsequently, the reaction was continued under stirring and reflux in a nitrogen atmosphere at 60 °C for an additional hour. The modified MTS was collected by centrifugation and washed with 10% NaCl solution.

The modified MTS were stirred in 100 mL of 10% NaCl for 20 min to complete iodide-to-chloride ion exchange, followed by centrifugation. Finally, the product was washed three times with distilled water and lyophilized to yield the CMTS.

### 2.5. Synthesis of SIONP

A total of 16.2 g of FeCl_3_·6H_2_O and 8.4 g of FeSO_4_·7H_2_O were dissolved in 100 mL of distilled water in a 500 mL three-necked flask using sonication. The mixture was stirred and heated under nitrogen atmosphere. Once the temperature reached 80 °C, ammonium hydroxide was slowly added to adjust the pH to 11. Subsequently, 50 mL of 0.6 mol/L sodium citrate solution was added, and additional NH_3_·H_2_O was used to maintain the pH at approximately 11 throughout the reaction. The mixture was continuously stirred at 80 °C for 1 h. After the reaction, magnetic nanoparticles were separated using a magnet and washed repeatedly with distilled water until the pH of the supernatant reached neutral. The purified nanoparticles were vacuum-dried to yield the SIONP.

### 2.6. Fourier Transform Infrared Spectroscopy (FT-IR) Characterization

FT-IR spectroscopy was employed to characterize MTS, MTS-N, and CMTS. Each sample was thoroughly mixed with KBr and ground into fine powder. The mixture was then pressed into a pellet and analyzed using an FT-IR spectrometer to characterize the functional groups of MTS, MTS-N, and CMTS, with characteristic absorption bands annotated in the FT-IR spectra.

### 2.7. Ninhydrin Reaction

The ninhydrin reaction was employed to verify and quantify amino groups on MTS and CMTS. For qualitative identification, 4 mg of MTS or CMTS was separately reacted with 1 mL of 10 mg/mL ninhydrin solution at 80 °C, and the resulting color changes were visually assessed. For quantitative analysis, 4 mg of MTS was reacted with ninhydrin solution at 80 °C, and absorbance at 570 nm was measured spectrophotometrically. Amino content was calculated using a standard curve generated from glutamic acid standard, which was reacted with ninhydrin solution under the same conditions as MTS.

### 2.8. Measurement of ζ-Potential and Surface Charge Distribution

The ζ-potential of collagen was measured using dynamic light scattering (DLS) equipment (Litesizer 500, Anton Paar, Graz, Austria). Prior to testing, collagen solutions (initial pH~4) were adjusted to pH 4.0, 5.0, 6.0, 7.0, 8.0, 9.0, 10.0, and 11.0 using 5 mol/L NaOH, with fine adjustments made using 1 mol/L HCl as needed.

To evaluate the effect of cationic modification on surface charge distribution, measurements were conducted on MTS, MTS-N and CMTS at pH 9.0. The relative percentage of positively charged particles was determined based on the ζ-potential distribution function.

### 2.9. Preparation of Magnetic Suspensions

Magnetic particles, including SIONP, MTS, and CMTS, were each suspended at a concentration of 20 mg/mL in either distilled water or collagen solution (both pre-adjusted to pH 9.0–9.5). The resulting suspensions were shaken 50 times and sonicated for 5 min to yield MTS aqueous suspension (MTS-AS), CMTS aqueous suspension (CMTS-AS), SIONP-collagen aqueous suspension (SIONP-Colas), MTS-collagen aqueous suspension (MTS-Colas), and CMTS-Colas. Unless otherwise specified, SIONP-Colas, MTS-Colas, and CMTS-Colas refer to samples prepared by fully dispersing SIONP, MTS, or CMTS in a collagen solution (pH 9.0–9.5) at a final concentration of 20 mg/mL.

### 2.10. Suspension Stability Test

MTS-AS, CMTS-AS, MTS-Colas, and CMTS-Colas suspensions at concentrations of 1, 10, and 20 mg/mL were prepared by dispersing the corresponding amounts of MTS or CMTS in distilled water or collagen solutions at a pH of 9.0. These suspensions were transferred to microcuvettes (path length = 10 mm), and their optical density (OD) at 480 nm was measured every 30 min for 8 h using a UV-Vis spectrophotometer (Cary 60 UV-Vis, Agilent Technologies, Santa Clara, CA, USA). Suspension stability was evaluated by calculating the ratio of OD values at each time point to the initial OD value.

Next, MTS-AS and CMTS-Colas were transferred to microcuvettes, stored in a standing position, and photographed at designated time points to visually assess suspension stability. After 10 days of standing storage, the samples were gently shaken 20 times to resuspend the particles, and photographs were taken to document the resuspension outcome.

### 2.11. Delivery Performance Evaluation

Delivery performance was evaluated using syringes equipped with 31G and 26G needles, respectively. The aspiration-injection success rate was assessed after complete suspension. Evaluation was based on the successful completion of the entire aspiration-injection process.

During aspiration, the syringe needle was vertically inserted to draw the suspension from the bottom of the container. The pulling motion (force and position) of the syringe plunger was kept consistent, with only minor adjustments to the needle position. If the suspension could not be fully aspirated into the syringe after repeated attempts, the attempt was recorded as a failure. Similarly, if the aspirated suspension could not be fully expelled from the syringe despite applying additional force or repeated attempts, the injection was considered a failure. The success rate was recorded over 15 trials for each needle size, and the entire procedure was independently repeated three times.

### 2.12. Aggregate Size Determination of MTS-Colas and CMTS-Colas

The aggregate size of magnetic particles in freshly prepared MTS-Colas and CMTS-Colas was determined using DLS equipment (detection range: 3.8 nm–100 µm). The hydrodynamic diameters, calculated by the instrument software, were reported as the aggregate size of the sample. Then, after 8 h of standing storage, the suspensions were manually resuspended, and the hydrodynamic diameters was re-evaluated.

### 2.13. Cell Culture and Cytotoxicity Evaluation

L929, HUVEC, H22, and HepG2 cells were cultured in complete DMEM (high-glucose DMEM with 10% FBS and 1% P/S). Cells were seeded in culture plates or flasks and incubated at 37 °C in a 5% CO_2_ incubator.

For cytotoxicity evaluation, collagen solution was pre-adjusted to pH 9.0–9.5, and sterilized SIONP, MTS, and CMTS were suspended in PBS at a final concentration of 20 mg/mL. Prior to cell seeding, L929 and HUVEC cells were washed with PBS, detached using trypsin, and collected by centrifugation. The resulting cell pellets were resuspended in complete DMEM to a final concentration of 2 × 10^5^ cells/mL. Subsequently, 2 mL of the suspension was seeded into each well of 6-well plates and cultured until the cells adhered and reached confluence.

Before treatment, the culture medium was replaced with fresh complete DMEM. To evaluate cytotoxicity following exposure, 0.1 mL of each solution or suspension was added to the respective wells. The groups were designated as follows: PBS (Control), collagen solution (Collagen), SIONP suspension (SIONP), MTS suspension (MTS), CMTS suspension (CMTS), and CMTS-Colas suspension (CMTS-Colas). An equal volume of complete DMEM without cells served as the blank. After 24 and 48 h of incubation, 100 µL of CCK-8 solution was added to each well, followed by 2–4 h of incubation with gentle shaking every 20 min. Then, 100 µL of the supernatant was transferred to a 96-well plate. OD values at 450 nm were measured using a microplate reader, and cell viability was calculated as follows:

Cell viability (%) = [(OD_sample_ − OD_mean of blank_)/(OD_mean of control_ − OD_mean of blank_)] × 100 (1)

### 2.14. Apoptosis and Efficiency Assessment of In Vitro Hyperthermia

To evaluate the early-stage apoptosis and hyperthermia efficiency induced by CMTS-Colas, H22 and HepG2 tumor cells were divided into the following groups: Control (PBS), Collagen^HF-AMF^ (collagen solution, pH 9.0–9.5), SIONP-Colas^HF-AMF^, CMTS-Colas^HF-AMF^, and CMTS-Colas (without AMF exposure). Each treatment involved mixing 0.9 mL of cell suspension (2 × 10^6^ cells/mL) with 0.1 mL of the designated sterile solution or suspension. Hyperthermia treatment for all HF-AMF-labeled groups was simultaneously treated using a HF-AMF generator at 120 kHz and 8000 A/m for 30 min. During the treatment, the temperature of samples was monitored using an infrared thermal imager (PTi120, Fluke, Everett, WA, USA). The CMTS-Colas^HF-AMF^ group maintained an average temperature of 40–45 °C, with peak temperatures remaining below 47 °C. After each heating session, cells were centrifuged, resuspended in fresh complete medium, and incubated for 12 h before the next cycle.

For apoptosis assessment, samples were subjected to two hyperthermia cycles and subsequently incubated for 12 h. Following incubation, cells were centrifuged to obtain cell pellets, which were then resuspended in staining buffer. The resuspended cells were stained with DAPI and Annexin V-FITC/PI, transferred to 96-well plates, and analyzed using a fluorescence microscope (IM-5FLD, OPTIKA, Ponteranica, Italy). Apoptotic cells were visualized by green (Annexin V-FITC) and red (PI) fluorescence, while total cells were visualized by blue (DAPI) fluorescence.

For hyperthermia efficiency evaluation, cells underwent three hyperthermia cycles, followed by a 12 h incubation. Cell viability was assessed via the CCK-8 assay, using complete DMEM as the blank group. Cell viability was calculated as previously described, and hyperthermia efficiency was determined using the following equation:

Hyperthermia efficiency (%) = 100 − Cell viability(2)

### 2.15. Quantification of CMTS Retained by Tumor Cells

To determine the amount of CMTS retained by tumor cells after incubation, 0.9 mL of culture medium (control) or 0.9 mL of H22 or HepG2 cell suspension (2 × 10^6^ cells/mL) was incubated with 0.1 mL of CMTS-Colas in 6-well plates for 24 h. Following incubation, a magnet was applied to remove CMTS that were not retained by the cells. The cells were then rinsed with 1 mL PBS, and magnetic separation was repeated three times to further eliminate unretained particles.

After washing, the cells were detached using a cell scraper, resuspended in 1 mL of culture medium, and centrifuged together with the control samples at 10,000× *g* for 5 min to remove the supernatant. The total iron content in each sample was quantified using inductively coupled plasma mass spectrometry (ICP-MS; NexSAR-NexION 2200G, PerkinElmer, Waltham, MA, USA). The percentage of CMTS retained by tumor cells was calculated based on the ratio of iron content in the cell samples to that of the control group.

### 2.16. Statistical Analysis

All experiments were performed in triplicate, and data are presented as mean or mean ± standard deviation (SD) from three independent experiments. Statistical significance was assessed using the *t*-test, with significance levels denoted as follows: ns (not significant, *p* > 0.05), * (*p* < 0.05), ** (*p* < 0.01), and *** (*p* < 0.001).

## 3. Results and Discussion

### 3.1. Cultivation of Magnetotactic Bacteria and Extraction of MTS

The MTS employed in this study were derived from AMB-1, one of the few magnetotactic bacterial strains that can be stably cultured. The culture of AMB-1 was performed as previously described, while the extraction of MTS was further optimized based on our earlier method [18].

TEM images of both aggregated and isolated AMB-1 cells are presented in Figure S1. A representative TEM image of MTS is shown in Figure S2a. The regular lattice fringes observed in the TEM images indicated that the MTS possessed a well-crystallized structure (Figure S2b). Additionally, the TEM images revealed that the extracted MTS retained an intact biological membrane, with the thickness of approximately 2 nm (Figure S2c). This membrane has a complex composition, primarily consisting of fatty acids, proteins, polysaccharides and phospholipids, and features surface functional groups, such as amino and carboxyl groups, originating from the hydrolysis of membrane [19,20]. Quantitative analysis indicated that the MTS contained approximately 8 µg of NH_2_ groups per mg of MTS. The endotoxin levels of MTS extracted from different batches ranged from 20 to 120 EU/mg, which was considered safe for intratumoral injection, with no acute toxicity or pyrogenic effects [21].

### 3.2. Characterization of CMTS

**Figure 2:**
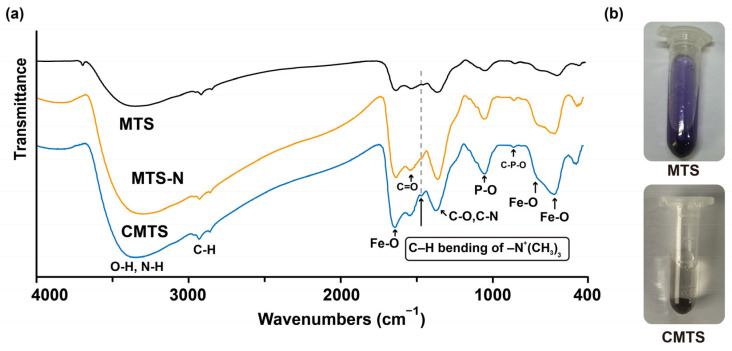
Characterization of CMTS. (**a**) FT-IR spectra of MTS, MTS-N, and CMTS, with dashed lines indicating the characteristic C–H bending peak of quaternized amine groups at 1465 cm^−1^. Additional FT-IR characteristic peaks corresponding to functional groups present on MTS and its modified forms are also labeled in the spectra; (**b**) Colorimetric comparison of MTS and CMTS following reaction with ninhydrin.

The cationic modification of the MTS surface followed the reaction pathway illustrated in Figure 1. The FT-IR spectrum (Figure 2a) of MTS reveals characteristic functional groups associated with the magnetic core and biological membrane, including Fe-O (580, 700 cm^−1^, 1600–1740 cm^−1^), P-O (1046 cm^−1^), C-O and C-N (1365 cm^−1^), C=O (1549 cm^−1^), C-H (2850–3000 cm^−1^), and -OH and -NH_2_ (3000–3650 cm^−1^) [22,23]. After modification, the characteristic peak corresponding to the carboxyl groups at 3710 cm^−1^ disappeared completely [24]. The FT-IR spectrum of the final product, CMTS, exhibited a characteristic peak at 1465 cm^−1^, corresponding to C–H bending in the quaternized amine groups, including both the original surface amines of MTS and those introduced during subsequent modification [25,26]. This peak is clearly visible in CMTS, whereas it is very weak in MTS and MTS-N, indicating the successful quaternization of surface amines on the magnetosomes. Additionally, the ninhydrin staining results further confirmed that the NH_2_ groups in MTS were fully reacted after modification (Figure 2b).

### 3.3. Stabilization Strategy and Preparation for CMTS-Colas

**Figure 3:**
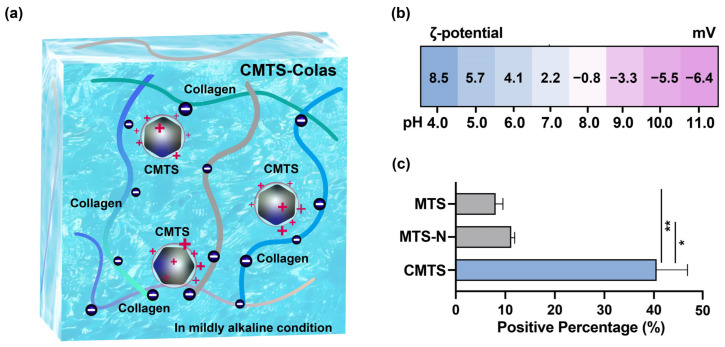
(**a**) Schematic diagram showing the formation of a stable suspension through electrostatic interactions between CMTS and collagen under mildly alkaline conditions; (**b**) Mean ζ-potential of collagen across a pH range of 4.0–11.0; (**c**) Distribution of relatively positively charged particles in MTS, MTS-N, and CMTS at pH 9.0. Data are presented as mean or mean ± SD; statistical significance is indicated as * (*p* < 0.05), ** (*p* < 0.01).

Despite cationic surface modification, CMTS suspensions exhibited significant sedimentation within 60 min at concentrations of 1 mg/mL, 10 mg/mL, and 20 mg/mL (Figure S3b), consistent with the previously reported suspension stability of poly-L-lysine-coated MTS [14]. To achieve prolonged stability of CMTS suspension, collagen was introduced as a stabilizing agent (Figure 3a). Collagen was chosen due to its extensive biomedical use, well-established processing methods, and abundance of amino acid residues, enabling pH-dependent tuning of the surface charge, thereby providing a negatively charged matrix to promote electrostatic interaction with the positively charged CMTS [27].

Collagen from different batches exhibited an initial pH of approximately 4–5, at which the ζ-potential ranged from +8.5 mV to +5.7 mV. As the pH increased to 6.0–7.0, the ζ-potential gradually decreased to +4.1 to +2.2 mV (Figure 3b). At pH 8.0, the ζ-potential shifted to −0.8 mV, indicating that the isoelectric point of collagen lies near pH 7–8—a range in which collagen becomes least stable and therefore unsuitable for subsequent formulation steps. When the pH further increased to 9.0–10.0, the ζ-potential decreased to −3.3 to −5.5 mV, reflecting a clearer tendency toward negative surface charge, consistent with the stability at this pH range.

Notably, Fe_3_O_4_ is expected unstable under acidic conditions, as H^+^ can induce iron dissolution and surface corrosion below pH 7.0 [28,29,30]. In contrast, OH^−^ catalyzes Fe_3_O_4_ formation without causing iron dissolution, making weakly alkaline conditions thermodynamically and chemically favorable for the stability of the MTS core [31,32,33].

The distribution of relatively positively charged particles for MTS, MTS-N, and CMTS at pH 9.0 was further analyzed (Figure 3c). The results showed that cationic modification increased the proportion of relatively positively charged particles from 8.2 ± 1.6% and 11.3 ± 0.7% to 40.7 ± 6.4%, indicating a significant enhancement in the positive surface charge distribution of CMTS. This also suggests that, under weakly alkaline conditions, CMTS can exhibit stronger charge interactions with collagen compared to native MTS.

Considering the effects of pH on the stability of the MTS core, as well as the ζ-potentials of collagen and positive surface charge of CMTS, a pH of 9.0–9.5 was selected for the CMTS-collagen aqueous suspension. The 0.5-unit range was set to accommodate minor variations during the pH adjustment of the collagen solution, thus minimizing operational complexity.

### 3.4. CMTS-Colas Showed Enhanced Stability at High Concentrations

**Figure 4:**
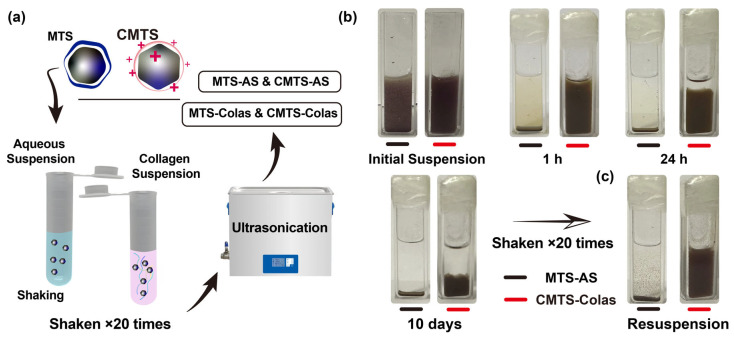
Enhanced suspension stability of CMTS-Colas. (**a**) Schematic illustration of the preparation procedures for MTS-AS, CMTS-AS, MTS-Colas, and CMTS-Colas; (**b**) Photographic comparison of the suspension stability of MTS-AS and CMTS-Colas under static conditions from initial dispersion up to 10 days; (**c**) Resuspendability of MTS-AS and CMTS-Colas after 10 days of static storage, evaluated by manual shaking.

The preparation of CMTS-Colas and other magnetosome-based suspensions is illustrated in Figure 4a. Spectroscopic analysis confirmed that a 1 mg/mL collagen solution effectively stabilized both MTS and CMTS suspensions at concentrations of 10 and 20 mg/mL for at least 8 h, with suspensions at 1 mg/mL maintaining over 80% stability after 8 h (Figure S3c,d). In contrast, MTS-AS and CMTS-AS at 1, 10, and 20 mg/mL exhibited significant sedimentation within 30 min to 1 h (Figure S3a,b).

As shown in the photographs, two formulations were compared at 20 mg/mL: MTS-AS, which represents the conventional and most widely used suspension form, and CMTS-Colas, which represents the enhanced stabilization strategy. The results clearly demonstrated the superior stability of CMTS-Colas, which remained well-suspended for at least 24 h, while MTS-AS exhibited nearly complete sedimentation within 1 h (Figure 4b). After 10 days of standing, around 60% of the suspension volume of CMTS-Colas had settled, but the remaining suspension retained a dispersed state, suggesting that CMTS-Colas exhibited good long-term stability at the high concentration of 20 mg/mL. To evaluate resuspension performance, the suspensions were manually shaken 20 times after 10 days of standing. CMTS-Colas rapidly returned to a homogeneous state, whereas MTS-AS failed to re-suspend effectively despite shaking (Figure 4c).

Previous studies have explored surface charge modification of MTS; however, suspension stability was typically maintained for less than one hour or was not extensively evaluated, with concentrations mostly limited to approximately 1 mg/mL or below [14,34,35]. In this study, introducing collagen as a negatively charged stabilizing matrix significantly enhanced suspension stability of magnetosome-based formulations, achieving sustained dispersion up to 20 mg/mL and providing a versatile method easily applicable to conventional MTS preparations.

### 3.5. CMTS-Colas Exhibited Improved Delivery Performance with Fine Needles

**Figure 5:**
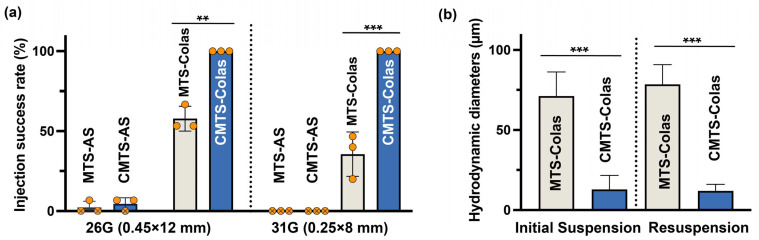
Enhanced syringe delivery performance of CMTS-Colas. (**a**) Delivery success rates of MTS-AS, CMTS-AS, MTS-Colas, and CMTS-Colas using 26G and 31G syringes, with the indicated dimensions referring to the outer diameters of the respective needle gauges; (**b**) Hydrodynamic diameters of MTS-Colas and CMTS-Colas immediately after dispersion and following 8 h of standing with manual resuspension. Statistical significance is indicated as ** (*p* < 0.01), *** (*p* < 0.001).

The syringe-based delivery performance of MTS-Colas and CMTS-Colas was evaluated using fine-gauge needles to assess their compatibility with standard injection practices. As shown in Figure 5a, CMTS-Colas achieved a 100% success rate for complete aspiration and expulsion through both 26G and 31G needles, whereas MTS-Colas exhibited significantly lower success rates of 57.8 ± 7.7% and 35.6 ± 13.9%, respectively. In comparison, MTS-AS and CMTS-AS suspensions (20 mg/mL)—aqueous dispersions without collagen—showed extremely low delivery efficiencies (<10%) with 26G needles and failed completely with finer ones. These results suggest that the introduction of collagen as a negatively charged stabilization matrix markedly enhances dispersion stability and injectability, and that combining cationic CMTS with collagen provides the most favorable performance for fine-needle delivery.

To elucidate the origin of this enhanced delivery behavior, we analyzed the aggregation characteristics of MTS-Colas and CMTS-Colas (Figure 5b). The hydrodynamic diameters of magnetic aggregates differed substantially: 71.2 ± 15.1 µm for MTS-Colas versus 12.8 ± 8.7 µm for CMTS-Colas in freshly prepared samples. After 8 h of standing storage followed by manual resuspension, these values remained relatively stable (78.5 ± 12.4 Μm vs. 12.0 ± 4.2 µm, respectively), indicating good dispersion retention over time. Considering that the inner diameters of 26G and 31G needles are approximately 0.26 mm (260 µm) and 0.13 mm (130 µm), respectively, the large aggregates of MTS-Colas could readily cause partial blockage or irregular flow during injection. In contrast, the smaller and more uniform aggregates of CMTS-Colas could pass smoothly through both needle types. This improved injectability can be attributed to the stronger electrostatic interactions between the cationic CMTS surfaces and the negatively charged collagen network, which maintain suspension stability and prevent clogging during injection.

Our findings are consistent with previous reports underscoring the importance of electrostatic stabilization in improving the injectability of magnetic colloidal systems. For instance, Wu et al. (2024) developed injectable magnetic hydrogels by assembling positively charged gelatin nanoparticles with negatively charged Fe_3_O_4_ nanoparticles coated with polyacrylic acid at high concentrations [36]. This charge-based assembly effectively reduced aggregation and improved injectability for minimally invasive thermal therapies, confirming the broad applicability of electrostatic interaction–driven stabilization strategies in magnetic formulations.

### 3.6. Cytocompatibility Evaluation of CMTS-Colas and Its Components

**Figure 6:**
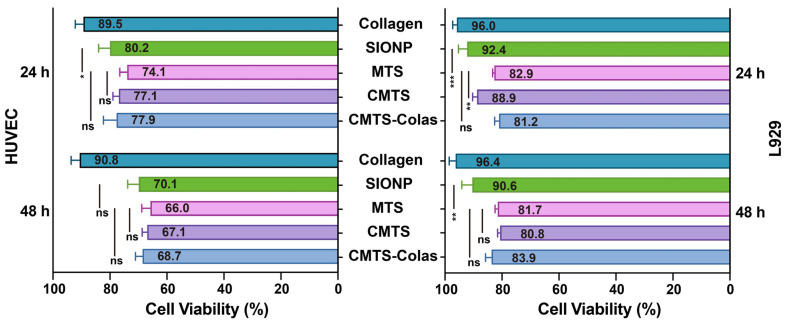
In vitro biocompatibility of SIONP, MTS, CMTS-Colas, and individual components of CMTS-Colas. Viability of L929 and HUVEC cell layers in 2 mL medium was assessed after 24 h or 48 h co-culture with different treatments: 0.1 mL collagen solution, 2 mg SIONP, 2 mg MTS, 2 mg CMTS, CMTS-Colas (containing 2 mg CMTS and 0.1 mL collagen solution at 1 mg/mL, pH 9.0–9.5). Statistical significance is indicated as ns (*p* > 0.05), * (*p* < 0.05), ** (*p* < 0.01), and *** (*p* < 0.001).

Although CMTS-Colas was developed for intratumoral injection, potential exposure to normal cells during administration necessitated a comprehensive evaluation of its cytocompatibility. As shown in Figure 6, the cell viability of 0.1 mL CMTS-Colas and its major components on HUVEC and L929 cell viability were assessed. The collagen solution reduced cell viability by less than 11%, indicating good biocompatibility. MTS exhibited higher cytotoxicity than SIONP, but the increased cytotoxicity remained within 10%.

When 2 mg of CMTS was co-cultured with HUVEC and L929 cells for 24 and 48 h, cell viability consistently remained above 60%, indicating comparable biocompatibility to MTS. For the complete CMTS-Colas formulation, HUVEC and L929 cell viabilities were 77.9% and 81.2% after 24 h, and 68.7% and 83.9% after 48 h, respectively. Overall, CMTS-Colas maintained acceptable cytocompatibility, comparable to that of the individual components.

In contrast, previous studies reported that surface coating of MTS with cationic materials led to greater cytotoxicity in 3T3 fibroblasts at 1 mg/mL [14]. These findings suggest that directly modifying the native –COOH and –NH_2_ groups on the magnetosome membrane may offer a more biocompatible alternative to traditional cationic coatings.

### 3.7. In Vitro Hyperthermia Performance of CMTS-Colas

**Figure 7:**
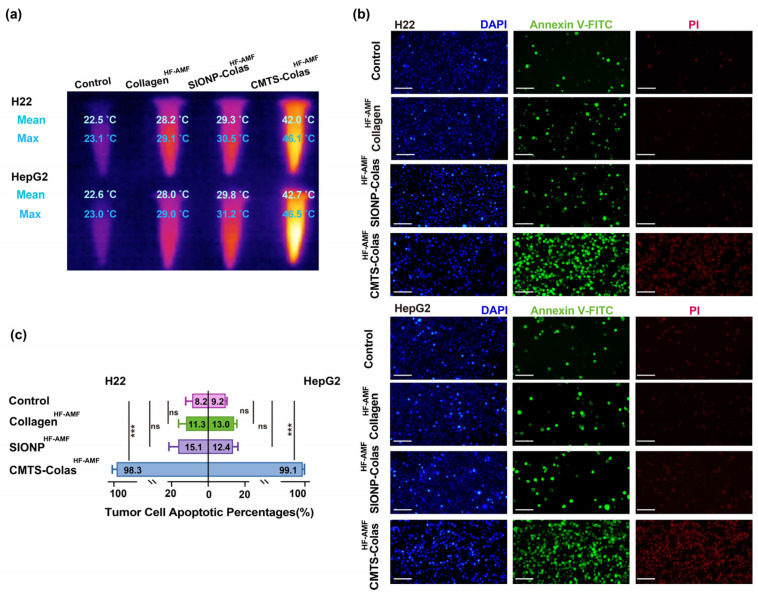
In vitro hyperthermia and apoptotic evaluation of CMTS-Colas. (**a**) Representative infrared thermal imaging of control, collagen^HF-AMF^, SIONP-Colas^HF-AMF^, and CMTS-Colas^HF-AMF^ induced by HF-AMF (120 kHz, 8000 A/m); (**b**) Fluorescence images of H22 and HepG2 cells after two sessions of hyperthermia (scale bar: 100 µm); (**c**) Apoptotic cell percentages quantified based on fluorescence images. Early apoptotic cells are indicated by green fluorescence (Annexin V–FITC), late apoptotic or necrotic cells are marked by red fluorescence (PI), and total cell nuclei are shown in blue (DAPI). Data are presented as mean ± SD. Statistical significance: ns (*p* > 0.05), *** *p* < 0.001.

Hyperthermia treatments were conducted using a HF-AMF generator. Hyperthermia temperatures were monitored, ensuring that the maximum thermal point did not exceed 47 °C and that the average remained within the therapeutic range of 40–45 °C. Collagen alone showed no heating under HF-AMF exposure, maintaining near-baseline temperatures. SIONP-Colas at 2 mg/mL failed to achieve therapeutic hyperthermia temperatures, which may be attributed to the suboptimal magnetic heating performance of SIONPs at 120 kHz and 8000 A/m [37]. In contrast, CMTS-Colas at the same concentration successfully elevated the temperature of 1 mL of culture medium to the therapeutic range of 40–45 °C, fulfilling the thermal requirements for hyperthermia therapy (Figure 7a).

To simulate clinical conditions where repeated hyperthermia sessions are often required for effective tumor suppression, H22 and HepG2 cells were subjected to two HF-AMF treatments [38,39]. Fluorescence staining (Figure 7b,c) showed apoptosis rates of approximately 8–9% in untreated controls, likely resulting from procedural cell loss during culture and staining [40], and slightly elevated rates (~11–13%) in the Collagen^HF-AMF^ group. SIONP-Colas^HF-AMF^ induced moderate apoptosis, with 15.1% in H22 and 12.4% in HepG2 cells, reflecting limited hyperthermia efficacy due to suboptimal heating. CMTS-Colas^HF-AMF^ induced apoptosis in over 98% of both cell types, demonstrating remarkable therapeutic efficacy through hyperthermia. However, a small fraction of tumor cells remained non-apoptotic, and given their proliferative nature, a potential risk of recurrence cannot be excluded, highlighting the necessity of multiple hyperthermia treatments.

**Figure 8:**
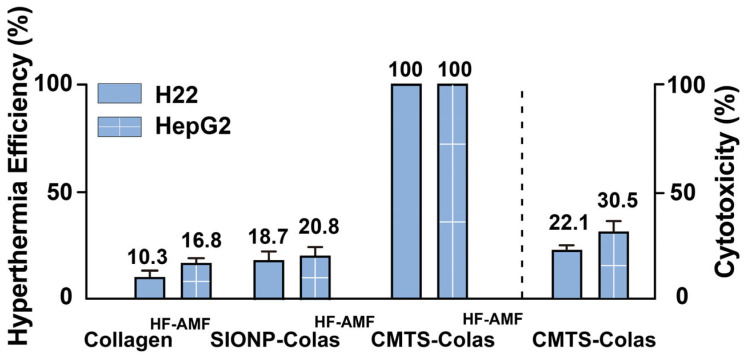
In vitro hyperthermia treatment efficacy in H22 and HepG2 cells after three treatment sessions. Data are presented as mean ± SD.

To further quantify hyperthermia efficiency, cell viability was assessed after three hyperthermia sessions (Figure 8). Collagen showed no significant hyperthermia effect, and SIONP-Colas induced mild inhibition, with cell viability reductions of less than 25%. CMTS-Colas combined with HF-AMF completely inhibited the viability of both H22 and HepG2 cells after three treatment cycles, confirming their hyperthermia efficacy. In the CMTS-Colas group without HF-AMF exposure, the intrinsic cytotoxicity of CMTS-Colas was 30.5% for HepG2 cells and 22.1% for H22 cells, indicating that the inhibition observed in the CMTS-Colas + HF-AMF group was attributable to hyperthermia rather than material-related toxicity.

Fe quantity measurements indicate that 10.9 ± 1.1% and 15.2 ± 2.1% of CMTS can be retained by H22 and HepG2 tumor cells. However, the relative proportions of CMTS that remain bound on the cell surface versus those that are internalized—and the specific subcellular compartments in which internalized CMTS accumulate—remain to be clarified. Such information would help determine how effectively heat generated by CMTS is transferred to thermally sensitive intracellular structures, thereby offering more precise insight into hyperthermia efficiency and the downstream biological responses. Moreover, while our in vitro experiments demonstrated robust therapeutic efficacy—showing that 2 mg of CMTS can inhibit 1 mL of tumor cell suspension after three rounds of magnetic hyperthermia—further comprehensive in vivo evaluation is required to validate the translational potential of this system.

## 4. Conclusions

This study presents a promising strategy for stabilizing magnetosomes through cationic surface modification of MTS to produce CMTS, combined with collagen as a biocompatible stabilizing matrix. To our knowledge, this represents the first application of collagen to enhance both suspension stability and injectability of magnetosome formulations, particularly with fine-gauge needles. The cationic modification process—through amination and subsequent quaternization—imparts a stable and significantly elevated positive surface charge to CMTS, especially under mildly alkaline conditions. By strengthening electrostatic interactions with negatively charged collagen, the increased surface charge helps achieve a smaller and more homogeneous particle size distribution, ultimately enhancing the injectability of the CMTS suspension.

Although CMTS alone does not exhibit markedly improved stability or injectability, the introduction of collagen serves as an effective strategy. As a flexible biomolecular matrix, negatively charged collagen forms electrostatic interactions with positively charged CMTS, effectively preventing aggregation without relying on high viscosity. This approach could inspire strategies to stabilize other charged and inherently unstable suspensions while enhancing their injectability.

Furthermore, the CMTS-Colas formulation enables straightforward sterile preparation and resuspension. CMTS-Colas allowed us to achieve a high-concentration formulation of 20 mg/mL that remained stable for at least 24 h under static conditions, representing a notable advantage for magnetic hyperthermia applications where high doses of magnetic materials are often required. This higher concentration reduces the required injection volume, enhancing feasibility. Even after 8 h of static storage, the suspension could be fully resuspended by manual shaking and successfully injected through a 31 G needle, demonstrating its good practical handling and usability. In addition to improvements in stabilization and delivery performance, CMTS-Colas exhibited strong heating efficiency during in vitro magnetic hyperthermia, indicating that the stabilization strategy does not compromise magnetic functionality and may further enhance therapeutic outcomes in future in vivo studies.

## Data Availability

The original contributions presented in the study are included in the article, further inquiries can be directed to the corresponding authors.

## Supplementary Materials

The following supporting information can be downloaded at: https://www.mdpi.com/article/10.3390/jfb16120461/s1, Figure S1: TEM images of *Magnetospirillum magneticum* AMB-1 in (a) an aggregated state and (b) an isolated state; Figure S2: (a) TEM image of magnetosomes. (b) Magnified view showing lattice fringes of the magnetic core. (c) Magnified view of the magnetosome membrane, pseudo-colored using the Lookup Tables (LUT) tool in ImageJ software (version 1.54j) to enhance visualization; Figure S3: Suspension stability of MTS-AS (a), CMTS-AS (b), MTS-Colas (c), and CMTS-Colas (d) at concentrations of 1, 10, and 20 mg/mL, evaluated over an 8 h sedimentation period using spectrophotometric measurements.

## Abbreviations

The following abbreviations are used in this manuscript:

MTS	Magnetosomes
CMTS	Cationized magnetosomes
CMTS-Colas	Cationized magnetosomes-collagen aqueous suspension
MHT	Magnetic hyperthermia therapy
HF-AMF	High-frequency alternating magnetic field
AMB-1	*Magnetospirillum magneticum* strain AMB-1
MTS-N	Amine-modified magnetosomes
MTS-AS	Magnetosomes aqueous suspension
CMTS-AS	Cationized magnetosomes aqueous suspension
SIONP-Colas	SIONP-collagen aqueous suspension
MTS-Colas	Magnetosomes-collagen aqueous suspension
