# Supplementary material for: Cationic Surface Modification Combined with Collagen Enhances the Stability and Delivery of Magnetosomes for Tumor Hyperthermia
Source: J Funct Biomater. 2025 Dec 12;16(12):461. doi: 10.3390/jfb16120461 (PMC12733886; doi:10.3390/jfb16120461)
Supplement: Supplementary file 1 [file jfb-16-00461-s001.zip › jfb-3991396-supplementary.pdf]

Journal of
Functional Biomaterials

MDPI

Article

Cationic Surface Modification Combined with Collagen Enhances the Stability and Delivery of Magnetosomes for Tumor Hyperthermia

Yu Wang [1,2,†], Conghao Lin [3,†], Yubing Zhang [4], Wenjun Li [1], Hongli Cui [1], Bohan Li [5], Zhengyi Liu [1], Kang Wang [1], Qi Wang [1], Yinchu Wang [1], Kangning Lv [6], Yandi Huang [7], Hongqin Zhuang [4,*] and Song Qin [1,*]

[1] Key Laboratory of Coastal Biology and Biological Resource Utilization, Yantai Institute of Coastal Zone Research, Chinese Academy of Sciences, Yantai 264003, China; wangyu209@mails.ucas.ac.cn (Y.W.); wjli@yic.ac.cn (W.L.); hlcui@yic.ac.cn (H.C.); zyliu@yic.ac.cn (Z.L.); kangwang@yic.ac.cn (K.W.); sdlywangqi@163.com (Q.W.); ycwang@yic.ac.cn (Y.W.)
[2] University of Chinese Academy of Sciences, Beijing 100049, China
[3] State Key Laboratory of Bioelectronics, National Demonstration Center for Experimental Biomedical Engineering Education, School of Biological Science and Medical Engineering, Southeast University, Nanjing 210096, China; 230258335@seu.edu.cn
[4] The State Key Laboratory of Pharmaceutical Biotechnology, School of Life Sciences, Nanjing University, Nanjing 210023, China; 602023300062@smail.nju.edu.cn
[5] Featured Laboratory for Biosynthesis and Target Discovery of Active Components of Traditional Chinese Medicine, School of Traditional Chinese Medicine, Binzhou Medical University, Yantai 264003, China; bohanli@bzmc.edu.cn
[6] State Key Laboratory of Heavy Oil Processing and Center for Bioengineering and Biotechnology, College of Chemistry and Chemical Engineering, China University of Petroleum (East China), Qingdao 266580, China; bz23030029@s.upc.edu.cn
[7] School of Life Sciences, Yantai University, Yantai 264003, China; 201970503202@s.ytu.edu.cn
* Correspondence: hqzhuang@nju.edu.cn (H.Z.); sqin@yic.ac.cn (S.Q.)
† These authors contributed equally to this work.

Supplementary Materials:

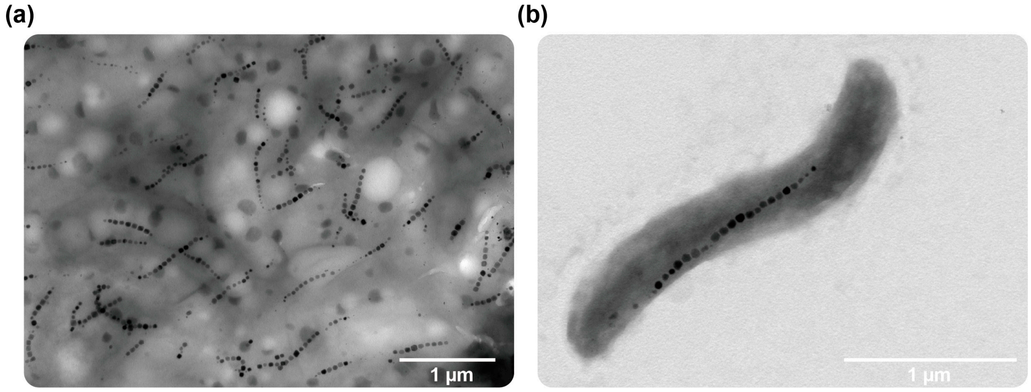

Figure S1. TEM images of *Magnetospirillum magneticum* AMB-1 in (a) an aggregated state and (b) an isolated state.

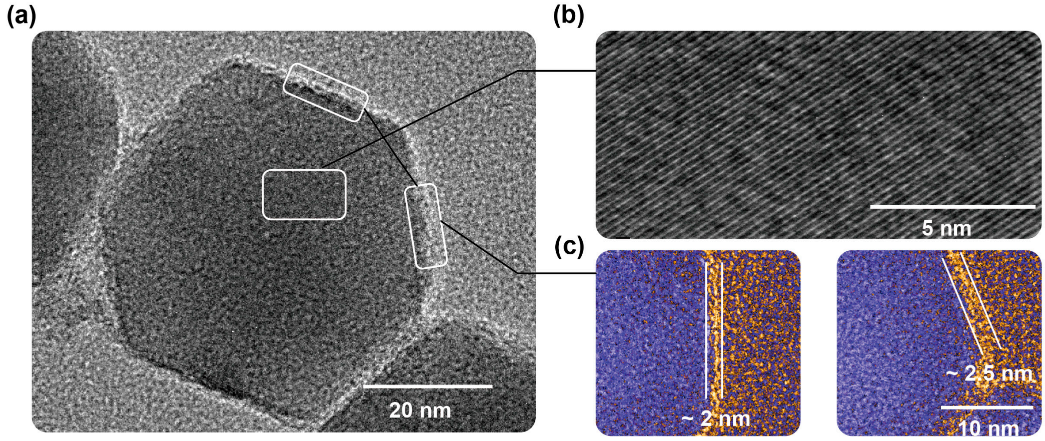

Figure S2. (a) TEM image of magnetosomes. (b) Magnified view showing lattice fringes of the magnetic core. (c) Magnified view of the magnetosome membrane, pseudo-colored using the Lookup Tables (LUT) tool in ImageJ software (version 1.54j) to enhance visualization.

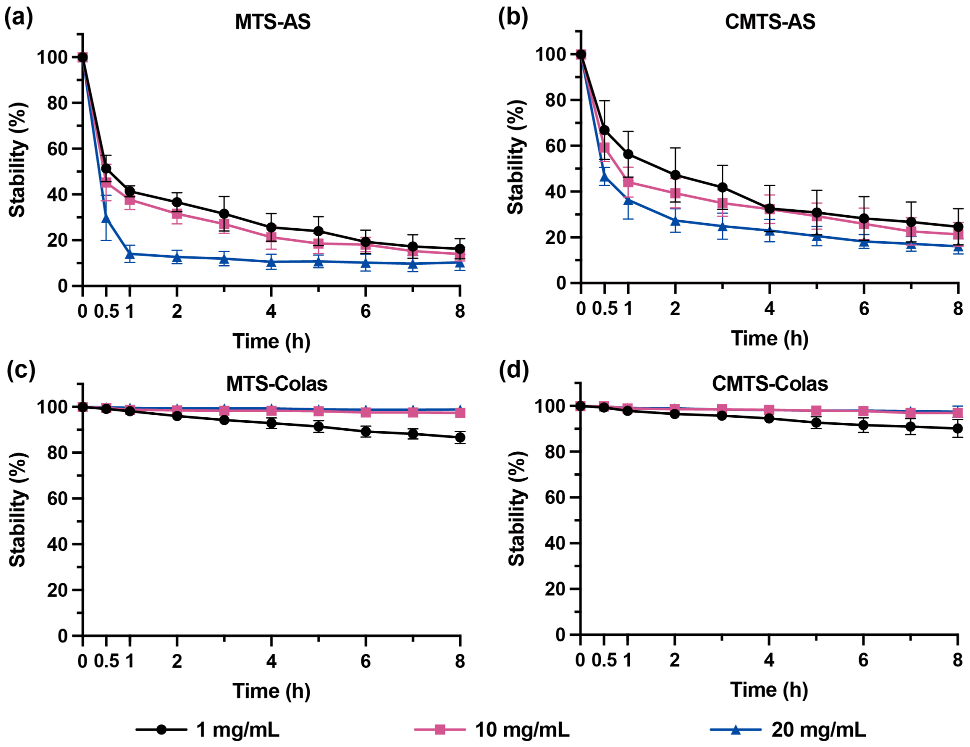

Figure S3. Suspension stability of MTS-AS (a), CMTS-AS (b), MTS-Colas (c), and CMTS-Colas (d) at concentrations of 1, 10, and 20 mg/mL, evaluated over an 8-hour sedimentation period using spectrophotometric measurements.
